# The Association between Metabolic Syndrome and Elevated Alanine Aminotransferase Levels in an Indigenous Population in Northern Taiwan: A Community-Based and Cross-Sectional Study

**DOI:** 10.1155/2020/6612447

**Published:** 2020-12-09

**Authors:** Yi-Fang Chen, Yen-An Lin, Wei-Chung Yeh, Yu-Chung Tsao, Wen-Cheng Li, Wei-Ching Fang, I-Ju Chen, Jau-Yuan Chen

**Affiliations:** ^1^Department of Family Medicine, Chang-Gung Memorial Hospital, Linkou Branch, Taoyuan City 33305, Taiwan; ^2^Department of Occupational Medicine, Chang Gung Memorial Hospital, Linkou Branch, Taoyuan City 33305, Taiwan; ^3^Chang Gung University College of Medicine, Taoyuan City 33302, Taiwan; ^4^Department of Health Management, Xiamen Chang-Gung Hospital, Xiamen 361028, China

## Abstract

Our study aims to determine the prevalence of metabolic syndrome (MetS) among the Northern Taiwanese indigenous population and to explore the relationship between MetS and liver enzyme, especially serum alanine transaminase (ALT). This is an observational and cross-sectional study that was conducted in remote villages of an indigenous community in Northern Taiwan between 2010 and 2015. MetS was defined based on the revised NCEP/ATPIII criteria from Taiwan Health Promotion Administration. A total of 454 participants were included in the analysis. There were 277 people with MetS and 177 people without. The prevalence of MetS was 61.01%. The average age was 49.50 years. People with MetS had a significantly higher liver enzyme (ALT) level than those without MetS. In addition, the study showed that participants with higher ALT had a tendency towards a higher prevalence of MetS (76.7% vs. 57.3%, *p* = 0.001). The adjusted odds ratio (OR) of ALT levels >36 U/L for MetS was 2.79 (95% CI = 1.24–6.27, *p* = 0.01). The area under the ROC curve (AUC) of the ALT level was 0.63 (95% CI = 0.58–0.68, *p* < 0.001), which showed that the ALT level was positively associated with MetS. The overall prevalence of MetS was 61.01% in the highland indigenous population in Northern Taiwan; this study indicated that higher serum ALT levels were associated with an increased risk of MetS.

## 1. Introduction

Metabolic syndrome (MetS), a serious global health concern, is a constellation of different metabolic abnormalities in an individual, including central obesity, hyperglycemia, hypertension, elevated triglyceride levels, and low high-density lipoprotein cholesterol levels [1]. These abnormalities result in an increased risk of cardiovascular events, type II diabetes, and chronic kidney disease [2, 3]. Therefore, the identification of risk factors associated with MetS is vital.

According to the Taiwan national survey, the Nutrition and Health Survey in Taiwan (NAHSIT), the prevalence of MetS increased from 13.6% (1993–1996) to 30% (2013–2016). Taking the regional and ethnic diversity in Taiwan into consideration, the NAHSIT 2005–2008 investigated the regional differences and showed that the indigenous area had the highest prevalence (52.1%) compared to the prevalence in the densely populated areas, the Offshore island and the Hakka area. Additionally, up to 71.6% of indigenous peoples were overweight or obese [4, 5]. Furthermore, two cross-sectional studies demonstrated a high prevalence of MetS among indigenous populations in Taiwan between 2010 and 2011 [6, 7]. Nevertheless, there are still limited studies on MetS among indigenous peoples in Taiwan. A hospital-based study showed the prevalence of metabolic syndrome in aboriginals in Southeastern Taiwan was 58.7%, with a higher prevalence (68.5%) in women than in men (50.3%) (*p* < 0.001) [6]. Thus, we decided to design a real world and community-based study to figure out whether the indigenous population in northern part of Taiwan has the same high prevalence or not. We intend to assess MetS status in an indigenous tribe, the Atayal tribe, which is widely distributed in the northern part of the Central Mountain Range of Taiwan.

Alanine transaminase (ALT) is an enzyme that mainly exists in hepatocytes, and when the liver is injured due to conditions such as viral hepatitis, ALT will be released, resulting in elevated serum ALT levels [8]. A large community study in Taiwan from 2003 to 2004 illustrated that the prevalence of elevated ALT in an adult population was 11.4%, and nonalcoholic fatty liver disease (NAFLD) seemed to be the most common cause of elevated ALT [9]. Moreover, there were 16.5% adults with abnormal ALT levels in an Atayal indigenous community [10]. Previous cross-sectional studies have reported a positive association between serum ALT level and MetS [11, 12]. A systemic review and meta-analysis and a large cohort study both showed that the increase in serum ALT levels increased the risk of MetS [13, 14]. In addition, the Taiwan indigenous groups have high prevalence of alcoholism, hepatitis B, and hepatitis C, and the prevalence of abnormal ALT level was also high in the Atayal tribe [10]. Therefore, our study aimed to determine the association and the prevalence of MetS with higher serum ALT levels among the Northern Taiwanese indigenous population.

## 2. Materials and Methods

### 2.1. Study Design and Study Population

**Figure 1:**
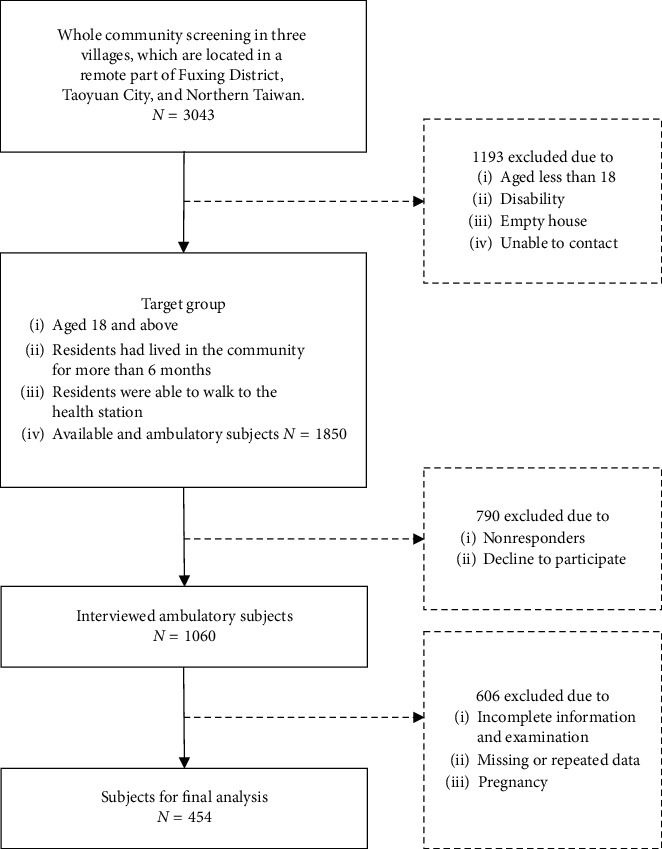
Flow chart of the study subjects.

This is an observational and cross-sectional study. Before this study, the minimum sample size for this study was calculated at the initial stage of the study. After previewing a relatively smaller population, considering 80% power, 95% confidence level, and 0.59 as the metabolic syndrome prevalence rate among indigenous population, we calculated that 276 participants were required to detect at least 2 odds ratios. We collected data from three remote villages in an indigenous community in Northern Taiwan from 2010 through 2015. Subjects were residents who had lived in the community for more than 6 months, aged over 18, and were able to walk to the health station. Every subject completed a questionnaire containing questions regarding basic personal data and medical conditions, including race, age, sex, occupation, and history of hypertension, diabetes, and hyperlipidemia. The questionnaires were completed after detailed explanations were provided by the trained interviewers in in-person interviews. Additionally, urine and blood samples were collected. Anthropometric measurements were performed by trained research assistants or nurses under the supervision of a medical doctor. After the exclusion of subjects who were pregnant or who had incomplete, missing, or repeated data, 454 participants were eligible for the analysis. The flow chart diagram (Figure 1) shows a gradual selection of individuals who comprised the subjects for final analysis. The study was approved by the Chang Gung Medical Foundation Institutional Review Board (99-0231B, 101-4156A3, 104-7978A3), and written informed consent was provided by all the subjects before enrollment.

### 2.2. Definition of Metabolic Syndrome and Abnormal ALT Level

MetS was defined by the revised NCEP/ATPIII criteria from Taiwan Health Promotion Administration [15]. In detail, a diagnosis of MetS requires the presence of three or more of the following factors: (1) waist circumference (WC) ≥90 cm in men or ≥80 cm in women, (2) systolic blood pressure (SBP) ≥130 mmHg or diastolic blood pressure (DBP) ≥85 mmHg or current use of antihypertensive drugs, (3) serum HDL-C <40 mg/dl in men or <50 mg/dl in women, (4) serum triglycerides ≥150 mg/dl or triglycerides-lowering drugs, and (5) fasting plasma glucose ≥100 mg/dl or current use of antihyperglycemic drugs. An abnormal ALT level was defined as a level >36 U/L according to the laboratory method used in Chang Gung Memorial Hospital.

### 2.3. Definition of Hypertension, Diabetes Mellitus, Hyperlipidemia, Smoking, and Alcohol Drinking

Hypertension was defined by a SBP ≥140 mmHg or DBP ≥90 mmHg or use of antihypertensive drugs. Diabetes mellitus was defined as a fasting plasma glucose ≥126 mg/dL, or glycated hemoglobin (HbA1c) ≥6.5, or the use of oral hypoglycemic agents or insulin therapy. Hyperlipidemia was defined as LDL-C ≥130 mg/dL, or triglycerides (TGs) ≥150 mg/dL, or total cholesterol (TC) ≥200 mg/dL, or use of lipid-lowering medications. Smoking was defined as at least 3 days a week in the recent month. Alcohol drinking habit was defined as more than 3 times a week.

### 2.4. Anthropometric and Laboratory Measurements

Each subject's blood pressure (BP) was measured on the upper arm while they were in a seated position after at least 5 minutes of rest with a standardized electronic sphygmomanometer. WC was measured at the level midway between the iliac crests and the lowest rib margin with the subjects in a standing position. Height was determined by a height-measuring machine while subjects were barefoot and standing in an erect position with their arms by their side and their feet together. Weight measurements were performed with subjects standing at the center of the weighing scale in light clothing without shoes or socks. Body mass index (BMI) was defined as the weight in kilograms divided by the square of the height in meters (kg/m^2^). Then, we classified the BMI values into 3 main categories according to the definition published by Taiwan Health Promotion Administration: (1) underweight (BMI < 18.5), (2) normal weight (18.5 ≤ BMI < 24), and (3) overweight or obesity (BMI ≥ 24) [16].

Blood samples were obtained from the antecubital vein after a 12 h overnight fast. All blood analyses were carried out at the clinical laboratory department of the Linkou Chang Gung Memorial Hospital, which is certified by the College of American Pathologists. Biochemical markers were analyzed on a Hitachi 7600–210 autoanalyzer (Hitachi, Tokyo, Japan); the biochemical markers included serum TC, HDL-C, TGs, fasting plasma glucose (FPG), ALT, high-sensitivity C-reactive protein (HS-CRP), HbA1c, and uric acid. In detail, the ALT levels were measured by a enzymatic method with a matched instrument application (Hitachi, Tokyo, Japan) in the Linkou Chang Gung Memorial Hospital.

### 2.5. Statistical Analysis

Data are presented as the mean and standard deviation for continuous variables or numbers and percentages for categorical variables. For variables with nonnormal distributions, the data are log-transformed for analysis and shown as median (interquartile range). In univariate analysis, independent sample *t*-tests and chi-square tests were used to compare MetS and non-MetS subjects. The chi-square test was also performed to determine the differences in two categorical variables and the prevalence of MetS in different serum ALT level groups. Correlations between different cardiometabolic risk factors and serum ALT levels were assessed with Pearson's correlation coefficient. The odds ratios (ORs) and their 95% confidence intervals (CIs) were determined for the association between cardiometabolic risk factors and MetS with adjustment for potential confounders, such as age, sex, alcohol drinking, and uric acid level. The receiver operating characteristic (ROC) curve was produced to acquire the values of the area under the curve (AUC) with 95% CIs and sensitivity and specificity values for the serum ALT level as a predictor of MetS. The analyses mentioned above were performed with SPSS Statistics version 22 (IBM, SPSS Armonk, NY, IBM Corp.). A *p* value of <5% was considered to indicate a statistically significant result.

## 3. Results

The baseline characteristics of the study population according to MetS diagnosis are presented in Table 1. A total of 454 participants were enrolled in the analysis, including 215 (47.36%) men and 239 (52.64%) women. There were 277 people with MetS and 177 people without. The prevalence of MetS was 61.01%. The average age was 49.50 years. People with MetS had a significantly higher ALT level (24.00 (17–36) U/L vs. 19.00 (15–26) U/L, *p* < 0.001) than those without MetS (Table 1). Also, the prevalence of antihypertensive, antilipidemic, and antidiabetic medication was 50% (86/172 = 50%), 23.48% (85/362 = 23.48%), and 44.58% (37/83 = 44.58%), respectively.

Furthermore, people with MetS had significantly higher levels of SBP, DBP, WC, BMI, FPG, serum HbA1c, TC, HS-CRP, TGs, and uric acid than those without MetS (Table 1). People with MetS were also older, more likely to be male, and had a higher prevalence of hypertension, diabetes, and hyperlipidemia but lower serum HDL-C levels. However, there were no statistically significant differences in the percentages of alcohol drinkers, smokers, and serum total bilirubin levels between the two groups.

**Figure 2:**
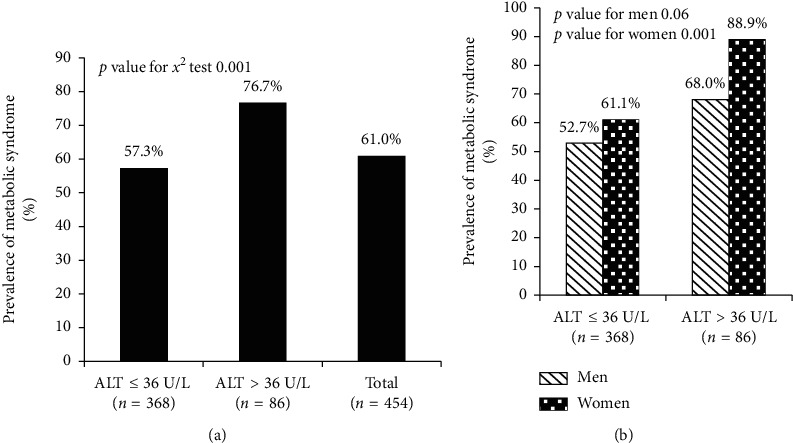
The comparisons of the prevalence of MetS in different ALT level groups and the total study group (a) and the prevalence of MetS of men and women in different ALT level groups (b).

The comparisons of the prevalence of MetS in different ALT levels >36 U/L and ≤36 U/L groups and the total study group are shown in Figure 2(a). The chi-square comparison showed that participants with ALT levels >36 U/L had a tendency towards a higher prevalence of MetS (76.7% vs. 57.3%, *p* = 0.001) compared with the prevalence of those with ALT levels ≤36 U/L. The bar chart illustrates that the ALT levels >36 U/L group had the highest MetS prevalence among the groups (Figure 2(b)). The chi-square comparison also showed that female participants with ALT levels >36 U/L had a tendency towards a higher prevalence of MetS (88.9% vs. 61.1%, *p* = 0.001) compared with the prevalence of those with ALT levels ≤36 U/L (Figure 2(b)).

The correlations between the serum ALT level and cardiometabolic risk factors are displayed in Table 2. The age-adjusted Pearson's coefficients of BMI, SBP, DBP, WC, FPG, and HS-CRP were 0.21 (*p* < 0.001), 0.14 (*p* = 0.01), 0.18 (*p* < 0.001), 0.26 (*p* < 0.001), 0.11 (*p* = 0.03), and 0.14 (*p* = 0.01), respectively, which indicated positive linear relationships with serum ALT levels (Table 2). The age-adjusted Pearson's coefficient of HDL-C was −0.12 (*p* = 0.02), which indicated a weak negative linear relationship with serum ALT levels. The serum Hb-1Ac, TC, TG, and uric acid levels had no statistically significant correlations with the serum ALT level.

The ORs of cardiometabolic risk factors for MetS are shown in Table 3. In the univariate logistic regression model, the crude ORs of ALT levels >36 U/L, each additional year of age, sex (men versus women), alcohol drinking (yes versus no), BMI ≧ 24 kg/m^2^, serum HS-CRP, and uric acid levels for MetS were 2.46 (95% CI = 1.43–4.22, *p* = 0.001), 1.04 (95% CI = 1.03–1.05, *p* < 0.001), 1.46 (95% CI = 1.00–2.13, *p* = 0.05), 1.36 (95% CI = 0.77–2.39, *p* = 0.29), 7.31 (95% CI = 4.72–11.32, *p* < 0.001), 1.04 (95% CI = 0.99–1.09, *p* = 0.14), and 1.25 (95% CI = 1.12–1.40, *p* < 0.001), respectively. In the univariate logistic regression model, the adjusted ORs of ALT levels >36 U/L, each additional year of age, sex (men versus women), alcohol drinking (yes versus no), BMI ≧ 24 kg/m^2^, serum HS-CRP, and uric acid levels for MetS were 2.79 (95% CI = 1.24–6.27, *p* = 0.01), 1.06 (95% CI = 1.04–1.08, *p* < 0.001), 2.88 (95% CI = 1.57–5.29, *p* = 0.001), 2.19 (95% CI = 1.09–4.41, *p* = 0.03), 9.40 (95% CI = 4.90–18.01, *p* < 0.001), 1.00 (95% CI = 0.94–1.05, *p* = 0.86), and 1.22 (95% CI = 1.02–1.45, *p* = 0.03), respectively (Table 3). The adjusted ORs of BMI < 18.5 kg/m^2^ for MetS were not statistically significant.

**Figure 3:**
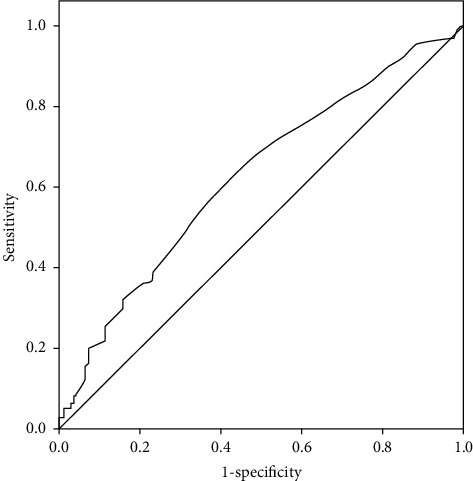
ROC curve and AUC for ALT as a predictor of MetS.

Finally, the AUC of the serum ALT level was 0.63 (95% CI = 0.58–0.68, *p* < 0.001), which showed that the serum ALT level was positively associated with MetS, and the cut-off point was 20.50 U/L (Table 4, Figure 3).

## 4. Discussion

The main finding of the present study is the high prevalence of MetS in a Taiwan indigenous population, and there was an association between serum ALT levels and MetS. Higher serum ALT levels, especially over 36 U/L, were associated with an increased risk of MetS.

The results were consistent with the findings of several previous studies. A hospital-based study showed that more than half of the indigenous adults in southeastern Taiwan had MetS [6], and two community-based studies revealed that the prevalence of MetS in indigenous populations in Northern Taiwan was over 48% [7, 17]. In contrast, without regard to regional difference, the data from the Taiwan Health Promotion Administration revealed that the overall prevalence of MetS in Taiwanese adults above the age of 20 was 19.7% in 2007 [15]. According to a cross-sectional survey, the MetS prevalence in a Taiwan metropolitan area was 33.32% among adults aged over 40 years in 2007 [18]. Therefore, it seems that the prevalence of MetS is higher in the Atayal tribe than in the metropolitan or overall population in Taiwan. This inference was in line with the results of the 2005–2008 NAHSIT, which revealed that the highest prevalence of MetS was found in the indigenous area (mountains) compared to the prevalence in other places in Taiwan [4]. Additionally, this idea was supported by a cross-sectional study illustrating that indigenous groups in Taiwan had a markedly higher prevalence of MetS than the Taiwanese and Hakka groups [19].

The reasons for the above phenomenon could be multifactorial. First, there were some health disparities between indigenous individuals and the general population in Taiwan [20]. For example, the lifetime prevalence of alcoholism according to ICD or DSM in four Taiwanese indigenous groups was 40–60% [21], and a large prospective cohort study revealed that heavy alcohol consumption is associated with an increased risk of the MetS [22]. In addition, a study suggested that 6% of inhabitants in Kaohsiung, the second-largest city of Taiwan, were current betel chewers, whereas 42% of the indigenous individuals aged over 15 years in southern Taiwan were current chewers [23]. Although areca nut chewing is deeply rooted in indigenous culture and symbolizes social belonging in Taiwan [24], chronic areca nut chewing is one of the independent risk factors for MetS and contributes to metabolic derangements via the involvement of tumor necrosis factor-*α*, leptin, and leukocyte count [25].

Second, there is a socioeconomic gap between the indigenous people and the general public in Taiwan. According to the economic status survey from the Council of Indigenous Peoples in Taiwan, the indigenous household income was approximately 61% of the average household income of Taiwan in 2014, and only 6.58% of primary income earners had a university education or above [26]. However, a study suggested that good socioeconomic status could protect against MetS [27], and a study with a large sample size also implied that a higher education level was related to a lower risk of MetS [28]. In other words, low socioeconomic status could negatively impact the health of indigenous people. Third, the indigenous population in our study was located in the mountains without adequate healthcare resources, and the lack of medical accessibility caused them to delay seeking care to improve their health outcomes. In summary, differences in health behaviors, low socioeconomic status, and limited access to healthcare in remote areas all impacted the health inequality between the Atayal tribe and individuals from metropolitan areas.

In the present study, the second major finding was that baseline serum ALT level and MetS were positively associated with the cut-off point of 20.50 U/L based on the AUC, and the adjusted OR of ALT levels >36 U/L for MetS was 2.79 after correction for age, sex, alcohol drinking, BMI, serum HS-CRP, and uric acid levels. These results corroborate the findings from a large-population, community-based study conducted in China, which verified a positive correlation between normal serum ALT levels and the morbidity rate of MetS after age and BMI correction. The optimal ALT boundary value based on the ROC curve was 24.5 for men and 14.5 U/L for women [29]. In short, an elevated ALT level, even at a level still within the reference interval, may reflect early metabolic changes.

On the other hand, our study also implied that an ALT level >36 U/L was associated with a tendency towards a higher prevalence of MetS compared to the prevalence associated with ALT levels ≤36 U/L. The finding was similar to the results of prior studies. A systematic review and meta-analysis revealed that the baseline circulating ALT level is associated with the risk of MetS and exhibits a linear dose-response relationship [14], which was identical to the results of a cross-sectional study including over 15000 adults and a longitudinal study with 7 years of follow-up from China [12, 13]. Taken together, these research studies have indicated that people with higher ALT levels have a higher risk of MetS.

In addition, a Korean study also confirmed that serum ALT levels were positively associated with MetS and its components (FPG, TGs, BP, and WC) [11], and we also found weak positive linear relationships between serum ALT levels and cardiometabolic risk factors (BMI, SBP, DBP, WC, FPG, and HS-CRP) in our study. Although the present study only included adults aged over 18, a nationwide study conducted in Iran indicated that MetS and some cardiometabolic risk factors were significantly associated with ALT levels in children and adolescents aged 7–18 years [30].

Although the mechanisms underlying the association between serum ALT levels and MetS are not entirely understood, a study from the Netherlands shed light on a possible mechanism. Insulin resistance acts as a major mediator of the association between the MetS and ALT level, while inflammatory adipokines, endothelial dysfunction, and nonesterified fatty acids also play minor roles but to a lesser extent [31]. ALT is a catalyzer that is involved in the transfer of the amino group of alanine to *α*-ketoglutarate [32], and a study from Argentina proposed that abnormal ALT levels are related to a dysregulation of normal amino acid metabolism in the liver, and aberrant liver metabolism could lead to MetS and insulin resistance [33]. Further studies are required to better clarify the pathophysiology.

## 5. Conclusions

In the present study, the overall prevalence of MetS was high in an indigenous population in Northern Taiwan, and higher serum ALT levels, especially those over 36 U/L, were associated with an increased risk of MetS. Further studies need to be performed with regard to this serious health problem in indigenous communities of Taiwan.

## Figures and Tables

**Table 1:** Study population characteristics according to the presence of MetS.

Variables	Total	Metabolic syndrome		*p* value
		No	Yes	
	(*n* = 454)	(*n* = 177)	(*n* = 277)	
Age (year)	49.50 ± 16.01	43.83 ± 16.97	53.12 ± 14.26	<0.001
SBP (mmHg)	133.03 ± 21.26	123.45 ± 18.21	139.15 ± 20.84	<0.001
DBP (mmHg)	83.80 ± 13.85	78.82 ± 12.53	86.97 ± 13.74	<0.001
WC (cm)	88.62 ± 11.00	81.23 ± 9.08	93.37 ± 9.40	<0.001
ALT (U/L)^a^	22.00 (16–32)	19.00 (15–26)	24.00 (17–36)	<0.001
FPG (mg/dL)^a^	93.00 (86–104.25)	87.00 (83–92)	99.00 (90–117)	<0.001
HbA1c (%)^a^	5.90 (5.6–6.2)	5.70 (5.5–5.9)	6.00 (5.7–6.5)	<0.001
HDL-C (mg/dL)	50.47 ± 13.05	56.40 ± 11.79	46.68 ± 12.41	<0.001
HS-CRP (mg/L)^a^	1.48 (0.72–2.94)	0.88 (0.4–2.2)	1.90 (0.96–3.52)	<0.001
TC (mg/dL)	194.28 ± 39.79	184.14 ± 33.21	200.75 ± 42.27	<0.001
Total bilirubin (mg/dL)^a^	0.50 (0.40–0.70)	0.50 (0.4–0.7)	0.50 (0.4–0.7)	0.24
TG (mg/dL)^a^	114.00 (80–183)	85.00 (62.0–115.5)	143.00 (103.5–215.0)	<0.001
Uric acid (mg/dL)	6.36 ± 1.88	5.91 ± 1.78	6.65 ± 1.88	<0.001
Men, *n* (%)	215 (47.36%)	94 (53.11%)	121 (43.68%)	0.0498

ALT				0.001
≦36 U/L	368 (81.06%)	157 (88.70%)	211 (76.17%)	
>36 U/L	86 (18.94%)	20 (11.30%)	66 (23.83%)	

BMI				<0.001
<18.5	10 (2.21%)	8 (4.55%)	2 (0.72%)	
18.5–24	152 (33.55%)	103 (58.52%)	49 (17.69%)	
≧24	291 (64.24%)	65 (36.93%)	226 (81.59%)	

HTN, *n* (%)	172 (38.05%)	35 (19.89%)	137 (49.64%)	<0.001
DM, *n* (%)	94 (20.70%)	5 (2.82%)	89 (32.13%)	<0.001
Hyperlipidemia, *n* (%)	362 (79.74%)	96 (54.24%)	266 (96.03%)	<0.001
Smoking, *n* (%)	43 (13.78%)	14 (12.28%)	29 (14.65%)	0.56
Alcohol drinking, *n* (%)	71 (22.61%)	22 (19.30%)	49 (24.50%)	0.29

Notes: clinical characteristics are presented as mean ± SD for continuous variables and *n* (%) for categorical variables. Variables with nonnormal distributions are log-transformed for analysis and shown as median (interquartile range). *p* values were derived from the independent *t*-test for continuous variables and chi-square test for categorical variables. ^a^ is considered as log-transformed variables. SBP, systolic blood pressure; DBP, diastolic blood pressure; WC, waist circumference; ALT, alanine aminotransferase; FPG, fasting plasma glucose; HbA1c, glycated hemoglobin; HDL-C, high-density lipoprotein cholesterol; HS-CRP, high-sensitivity C-reactive protein; TC, total cholesterol; TG, triglyceride; BMI, body mass index; HTN, hypertension; DM, diabetes mellitus.

**Table 2:** The correlations between ALT and cardiometabolic risk factors.

Variables	ALT U/L (*n* = 454)			
	Unadjusted		Adjusted for age	
	Pearson's coefficient	*p* value	Pearson's coefficient	*p* value
Age (year)	−0.07	0.147	NA	NA
BMI (kg/m^2^)	0.24	<0.001	0.21	<0.001
SBP (mmHg)	0.11	0.02	0.14	0.01
DBP (mmHg)	0.16	<0.001	0.18	<0.001
WC (cm)	0.26	<0.001	0.26	<0.001
FPG (mg/dL)	0.09	0.04	0.11	0.03
HbA1c (%)	0.08	0.08	0.09	0.10
HDL-C (mg/dL)	−0.12	0.01	−0.12	0.02
HS-CRP (mg/L)	0.15	0.001	0.14	0.01
TC (mg/dL)	0.02	0.60	0.01	0.82
Total bilirubin (mg/dL)	0.19	<0.001	0.23	<0.001
TG (mg/dL)	0.11	0.02	0.09	0.09
Uric acid (mg/dL)	0.10	0.03	0.10	0.05

ALT, alanine aminotransferase; BMI, body mass index; SBP, systolic blood pressure; DBP, diastolic blood pressure; WC, waist circumference; FPG, fasting plasma glucose; HbA1c, glycated hemoglobin; HDL-C, high-density lipoprotein cholesterol; HS-CRP, high-sensitivity C-reactive protein; TC, total cholesterol; TG, triglyceride.

**Table 3:** Univariate and multivariate logistic regression on the cardiometabolic risk factors related to MetS among the study population.

Variables	Crude odds ratio	(95% CI)	*p* value	Adjusted odds ratio	(95% CI)	*p* value
ALT (>36 U/L versus ≦36 U/L)	2.46	(1.43–4.22)	0.001	2.79	(1.24–6.27)	0.01
Age (year)	1.04	(1.03–1.05)	<0.001	1.06	(1.04–1.08)	<0.001
Sex (men versus women)	1.46	(1.00–2.13)	0.05	2.88	(1.57–5.29)	0.001
Alcohol drinking (yes versus no)	1.36	(0.77–2.39)	0.29	2.19	(1.09–4.41)	0.03

BMI (kg/m^2^)						
<18.5	0.53	(0.11–2.57)	0.43	1.53	(0.19–12.32)	0.69
18.5–24	1.00	—	—	1.00	—	—
≧24	7.31	(4.72–11.32)	<0.001	9.40	(4.90–18.01)	<0.001

HS-CRP (mg/L)	1.04	(0.99–1.09)	0.14	1.00	(0.94–1.05)	0.86
Uric acid (mg/dL)	1.25	(1.12–1.40)	<0.001	1.22	(1.02–1.45)	0.03

ALT, alanine aminotransferase; BMI, body mass index; HS-CRP, high-sensitivity C-reactive protein.

**Table 4:** The areas under ROC curve (AUC) and sensitivity, specificity by the optimized cut-off points for ALT in predicting metabolic syndrome.

Variables	AUC (95% CI)	*p* value	Cut-off point	Sensitivity	Specificity
ALT (U/L)	0.63 (0.58–0.68)	<0.001	20.500	0.643	0.559

ALT, alanine aminotransferase; ROC curve, receiver operating characteristic curve; CI, confidence interval.

## Data Availability

The data used to support the findings of this study are available from the corresponding author upon request.

## Abbreviations

MetS: Metabolic syndrome
NCEP: National Cholesterol Education Program Expert Panel
ATP III: Adult treatment panel III
NAFLD: Nonalcoholic fatty liver disease
SBP: Systolic blood pressure
DBP: Diastolic blood pressure
WC: Waist circumference
ALT: Alanine aminotransferase
FPG: Fasting plasma glucose
HbA1c: Glycated hemoglobin
HDL-C: High-density lipoprotein cholesterol
HS-CRP: High-sensitivity C-reactive protein
TC: Total cholesterol
TG: Triglyceride
BMI: Body mass index
HTN: Hypertension
DM: Diabetes mellitus.

## Additional Points

*Strengths and Limitations.* A key strength of this study is that to the best of our knowledge, there were few community-based studies demonstrating the association between serum ALT levels and MetS, especially targeting the Northern Taiwan indigenous population. Furthermore, it demonstrated high prevalence of MetS and the positive association between baseline serum ALT levels and MetS in the Taiwan Atayal tribe. Accordingly, the result of the study enhanced the knowledge of the Northern Taiwan indigenous population in public health scopes and may improve the indigenous health-care in clinical practice. There were also four limitations in this study: first, the cross-sectional design inherently limited the ability to effectively determine the causal relationship between MetS and serum ALT levels. Second, the serum ALT is a sensitive marker for liver dysfunction and affected by heavy alcohol consumption [34] and some medications [35], but some of the study population had several risk factors, including hypertension and dyslipidemia. Hence, we could not eliminate the possible effect of underlying diseases and medications used for these diseases on the present findings. There was also no clear personal history for viral hepatitis and NAFLD. Third, there were more female subjects than male subjects. The participants in the study were from three remote villages of Taoyuan City and had lived in these villages for over 6 months. The shift from rural low-wage labor to metropolitan higher-paying jobs reduced the available number of indigenous men in the mountain region. Fourth, there are selection bias and limited external validity in the study because the data were originated from 3 villages. Our study population just represented highland indigenous population in the northern part of Taiwan. Therefore, more comprehensive and meticulous consideration should be used in future studies. Further prospective population-based studies are needed to investigate the mechanisms in order to improve the above problems.
